# (Cyclo­butane-1,1-di­carboxyl­ato-κ^2^ *O*,*O*′)(1,10-phenanthroline-κ^2^ *N*,*N*′)platinum(II) dihydrate

**DOI:** 10.1107/S1600536813013378

**Published:** 2013-05-18

**Authors:** Pavel Štarha, Zdeněk Trávníček

**Affiliations:** a Department of Inorganic Chemistry, Faculty of Science, Palacký University, 17. listopadu 12, CZ-771 46 Olomouc, Czech Republic

## Abstract

The title compound, [Pt(C_6_H_6_O_4_)(C_12_H_8_N_2_)]·2H_2_O, which crystallizes as two independent formula units, has the metal atom in a square-planar geometry defined by two O atoms of the chelating cyclo­butane-1,1-di­carboxyl­ate dianion and two N atoms of the chelating 1,10-phenanthroline mol­ecule (r.m.s. deviations of the PtO_2_N_2_ units = 0.026 and 0.026 Å). Adjacent complex and water mol­ecules are connected through inter­molecular O—H⋯O hydrogen bonds and C—H⋯O, C⋯O [shortest C⋯O distance = 3.140 (5) Å], π–π [shortest C⋯C distances = 3.234 (6) and 3.347 (6) Å] and Pt⋯π [shortest Pt⋯C distance = 3.358 (4) Å] inter­actions into a three-dimensional network.

## Related literature

For platinum(II) cyclo­butane-1,1-di­carboxyl­ate complexes of other bidentate heterocyclic *N*-donor ligands, see: Ferreira *et al.* (1997 ▶); Yoo *et al.* (1999 ▶); Tu *et al.* (2003 ▶, 2004 ▶).
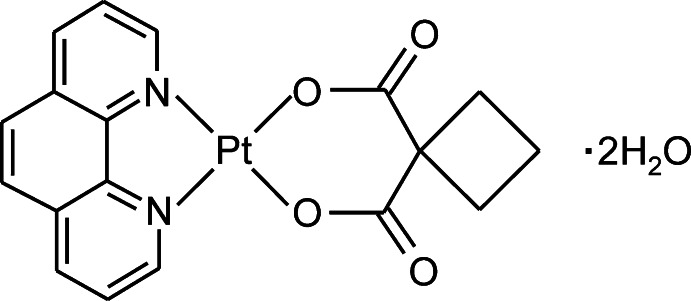


## Experimental

### 

#### Crystal data

[Pt(C_6_H_6_O_4_)(C_12_H_8_N_2_)]·2H_2_O
*M* *_r_* = 553.43
Triclinic, 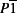

*a* = 10.93439 (14) Å
*b* = 11.83205 (18) Å
*c* = 13.51039 (19) Å
α = 84.7158 (12)°
β = 84.3918 (11)°
γ = 85.6240 (11)°
*V* = 1728.17 (4) Å^3^
*Z* = 4
Mo *K*α radiation
μ = 8.16 mm^−1^
*T* = 100 K
0.30 × 0.30 × 0.25 mm

#### Data collection

Agilent Xcalibur Sapphire2 diffractometer
Absorption correction: multi-scan (*CrysAlis PRO*; Agilent, 2012 ▶) *T*_min_ = 0.193, *T*_max_ = 0.235
13766 measured reflections
6042 independent reflections
5691 reflections with *I* > 2σ(*I*)
*R*_int_ = 0.019

#### Refinement

*R*[*F* ^2^ > 2σ(*F* ^2^)] = 0.021
*wR*(*F* ^2^) = 0.058
*S* = 1.12
6042 reflections
519 parameters
8 restraints
H atoms treated by a mixture of independent and constrained refinement
Δρ_max_ = 1.27 e Å^−3^
Δρ_min_ = −0.89 e Å^−3^

### 

Data collection: *CrysAlis PRO* (Agilent, 2012 ▶); cell refinement: *CrysAlis PRO*; data reduction: *CrysAlis PRO*; program(s) used to solve structure: *SHELXS97* (Sheldrick, 2008 ▶); program(s) used to refine structure: *SHELXL97* (Sheldrick, 2008 ▶); molecular graphics: *DIAMOND* (Brandenburg, 2011 ▶); software used to prepare material for publication: *publCIF* (Westrip, 2010 ▶).

## Supplementary Material

Crystal structure: contains datablock(s) I, global. DOI: 10.1107/S1600536813013378/ng5330sup1.cif

Structure factors: contains datablock(s) I. DOI: 10.1107/S1600536813013378/ng5330Isup2.hkl

Additional supplementary materials:  crystallographic information; 3D view; checkCIF report

## Figures and Tables

**Table 1:** Hydrogen-bond geometry (Å, °)

*D*—H⋯*A*	*D*—H	H⋯*A*	*D*⋯*A*	*D*—H⋯*A*
O5—H5*W*⋯O6	0.84	1.97	2.802 (5)	170
O6—H6*W*⋯O3	0.83	2.03	2.842 (4)	165
O7—H7*W*⋯O3*A*	0.83	1.93	2.753 (4)	169
O8—H8*V*⋯O7	0.85	1.95	2.806 (5)	175
O5—H5*V*⋯O8^i^	0.85	1.98	2.821 (5)	169
O6—H6*V*⋯O4^ii^	0.84	2.00	2.839 (4)	173
O7—H7*V*⋯O4*A* ^iii^	0.83	2.00	2.828 (4)	176
O8—H8*W*⋯O1^iv^	0.84	2.43	3.125 (4)	142
O8—H8*W*⋯O3^iv^	0.84	2.17	2.971 (4)	160
C7*A*—H7*AA*⋯O3^iv^	0.95	2.55	3.376 (5)	146
C7—H7*A*⋯O7^iv^	0.95	2.49	3.145 (5)	126
C8—H8*A*⋯O5^v^	0.95	2.59	3.259 (6)	128
C9—H9*A*⋯O6^v^	0.95	2.51	3.436 (6)	165
C14*A*—H14*A*⋯O8^vi^	0.95	2.35	3.230 (6)	154
C14—H14*B*⋯O5^vii^	0.95	2.36	3.309 (6)	174
C18*A*—H18*A*⋯O4*A* ^v^	0.95	2.38	3.196 (5)	144
C18—H18*B*⋯O4^v^	0.95	2.55	3.151 (5)	121

Symmetry codes: (i) 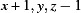; (ii) 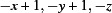; (iii) 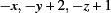; (iv) 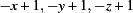; (v) 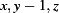; (vi) 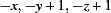; (vii) 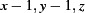.

**Fig. 1.**
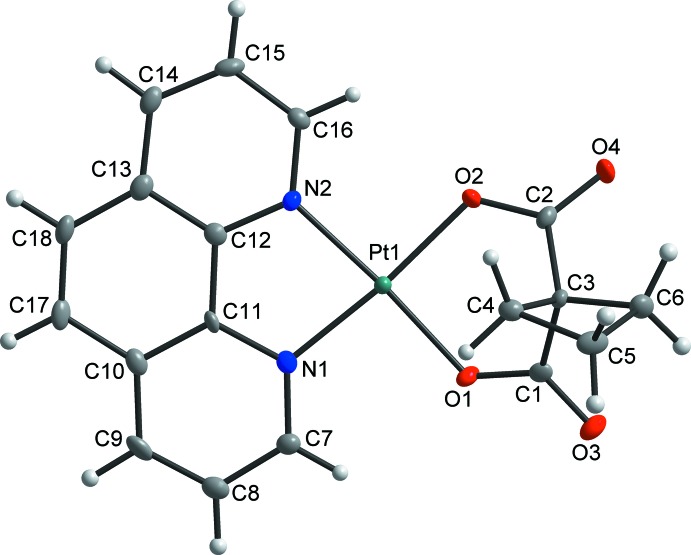
The molecular structure (one of two independent molecules within the asymmetric unit) of the title complex with the non-hydrogen atoms depicted as thermal ellipsoids at the 50% probability level.

**Fig. 2.**
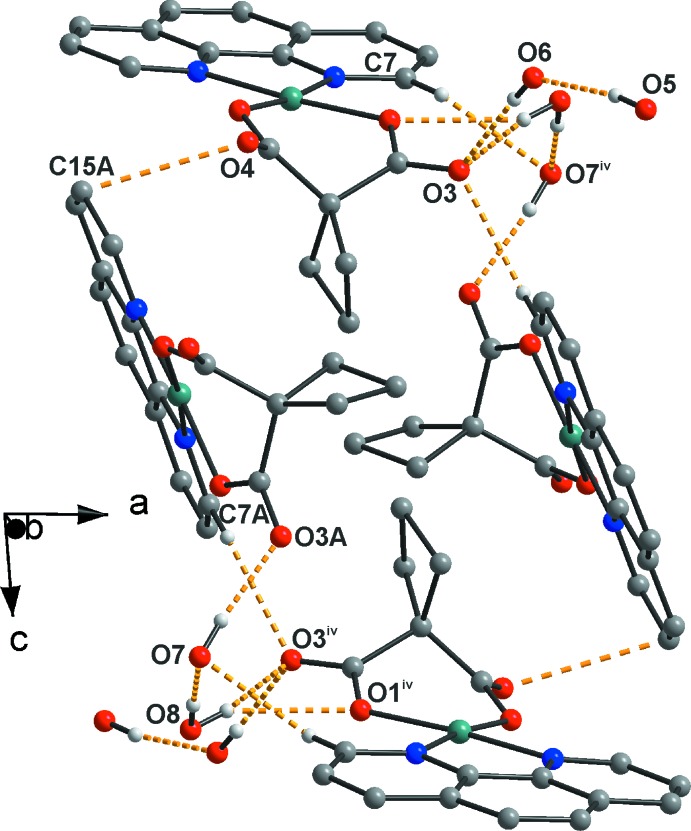
Part of the crystal structure of (I) showing the selected O5—H5W···O6, O6—H6W···O3, O7—H7W···O3A, O8—H8V···O7, O8—H8W···O1iv and O8—H8W···O3iv hydrogen bonds, and C7—H7A···O7iv (D—H, H···A, D···A (Å)and <DHA (°): 0.95, 2.49, 3.145 (6), 126), C7A—H7AA···O3iv (0.95, 2.55, 3.376 (6), 146) and C15A···O4 (D···A (Å): 3.140 (5)) non-covalent contacts (dashed orange lines); symmetry code: iv) –x+1, –y+1, –z+1; (see Table 1 for the parameters of O—H···O hydrogen bonds).

## supplementary crystallographic information

### Comment

The asymmetric unit of the title complex, [Pt(cbdc)(phen)].2H_2_O, (I), (Figure 1), contains two independent molecules of the complex and four water molecules of crystallization. The Pt^II^ atom is four-coordinated by two oxygen (cyclobutane-1,1-dicarboxylate dianion; cbdc) and two nitrogen (1,10-phenathroline; phen) atoms. The geometry is distorted square-planar with the N–Pt–O angles equalled to 92.64 (12)° and 93.57 (12)° for Pt1-molecule, and 93.18 (11)° and 93.25 (12)° for Pt2-molecule. The planes fitted through the Pt1O_2_N_2_ (r.m.s. deviation 0.026 Å) and Pt2O_2_N_2_ (r.m.s. deviation 0.026 Å) units form the dihedral angle of 61.42 (8)°, while the dihedral angle formed by both phen molecules is 63.34 (4)°. This corresponds with nearly coplanar orientation of the phen molecule and PtO_2_N_2_ unit (a dihedral angle of 3.57 (7)° for Pt1-molecule, and 2.58 (8)° for Pt2-molecule). The crystal structure contains O—H···O hydrogen bonds and C—H···O, C···O, π–π (the shortest C···C distances equal 3.234 (6) Å for C8···C16^viii^ and 3.347 (6) Å for C8A···C16A^vi^; symmetry codes: viii) –x+1, –y, –z; vi) –x, –y+1, –z+1) and Pt···π (the shortest Pt···C distance equals 3.358 (4) Å for Pt1···C10^viii^) types of the non-covalent contacts, which connect the molecules into a three-dimensional architecture (Figure 2, Table 1). The Pt1···Pt2, Pt1···Pt1^viii^ and Pt2···Pt2^vi^ distances are 7.8526 (2) Å, 5.2788 (2) Å, and 4.9992 (2) Å, respectively.

### Experimental

Warm (60 °C) distilled-water solutions of [Pt(cbdc)(dmso)_2_] (0.25 mmol) and 1,10-phenanthroline (0.25 mmol) were mixed together and stirred at 60 °C for 24 h. After that, the mixture was filtered and left to crystallize. The crystalline product, which formed in two weeks, was filtered off and washed with distilled water and methanol. Several crystals were collected for a single-crystal X-ray analysis. CH&N analysis calculated for C_18_H_18_N_2_O_6_Pt_1_: C 39.1, H 3.3, N 5.1%; found: C 39.2, H 3.3, N 5.3%. Elemental analysis (CH&N) was performed on a Thermo Scientific Flash 2000 CHNO-S Analyzer.

### Refinement

Non-hydrogen atoms were refined anisotropically and hydrogen atoms were located in difference maps and refined using the riding model with C—H = 0.95 (CH), C—H = 0.99 (CH_2_) Å, with *U*_iso_(H) = 1.2*U*_eq_(CH, CH_2_). The maximum and minimum residual electron density peaks of 1.27 and -0.89 e Å^-3^ were located 0.94 Å, and 0.78 Å from the Pt1, and Pt2 atoms, respectively.

### Crystal data

**Table:**

[Pt(C_6_H_6_O_4_)(C_12_H_8_N_2_)]·2H_2_O	*Z* = 4
*M**_r_* = 553.43	*F*(000) = 1064
Triclinic, *P*1	*D*_x_ = 2.127 Mg m^−^^3^
Hall symbol: -P 1	Mo *K*α radiation, λ = 0.71073 Å
*a* = 10.93439 (14) Å	Cell parameters from 17004 reflections
*b* = 11.83205 (18) Å	θ = 3.0–31.9°
*c* = 13.51039 (19) Å	µ = 8.16 mm^−^^1^
α = 84.7158 (12)°	*T* = 100 K
β = 84.3918 (11)°	Prism, yellow
γ = 85.6240 (11)°	0.30 × 0.30 × 0.25 mm
*V* = 1728.17 (4) Å^3^	

### Data collection

**Table:**

Agilent Xcalibur Sapphire2 diffractometer	6042 independent reflections
Radiation source: Enhance (Mo) X-ray Source	5691 reflections with *I* > 2σ(*I*)
Graphite monochromator	*R*_int_ = 0.019
Detector resolution: 8.3611 pixels mm^-1^	θ_max_ = 25.0°, θ_min_ = 3.0°
ω scans	*h* = −12→11
Absorption correction: multi-scan (*CrysAlis PRO*; Agilent, 2012)	*k* = −14→14
*T*_min_ = 0.193, *T*_max_ = 0.235	*l* = −14→16
13766 measured reflections	

### Refinement

**Table:**

Refinement on *F*^2^	Primary atom site location: structure-invariant direct methods
Least-squares matrix: full	Secondary atom site location: difference Fourier map
*R*[*F*^2^ > 2σ(*F*^2^)] = 0.021	Hydrogen site location: inferred from neighbouring sites
*wR*(*F*^2^) = 0.058	H atoms treated by a mixture of independent and constrained refinement
*S* = 1.12	*w* = 1/[σ^2^(*F*_o_^2^) + (0.0336*P*)^2^ + 3.750*P*] where *P* = (*F*_o_^2^ + 2*F*_c_^2^)/3
6042 reflections	(Δ/σ)_max_ = 0.002
519 parameters	Δρ_max_ = 1.27 e Å^−^^3^
8 restraints	Δρ_min_ = −0.89 e Å^−^^3^

### Special details

**Table:**

Geometry. All e.s.d.'s (except the e.s.d. in the dihedral angle between two l.s. planes) are estimated using the full covariance matrix. The cell e.s.d.'s are taken into account individually in the estimation of e.s.d.'s in distances, angles and torsion angles; correlations between e.s.d.'s in cell parameters are only used when they are defined by crystal symmetry. An approximate (isotropic) treatment of cell e.s.d.'s is used for estimating e.s.d.'s involving l.s. planes.
Refinement. Refinement of *F*^2^ against ALL reflections. The weighted *R*-factor *wR* and goodness of fit *S* are based on *F*^2^, conventional *R*-factors *R* are based on *F*, with *F* set to zero for negative *F*^2^. The threshold expression of *F*^2^ > σ(*F*^2^) is used only for calculating *R*-factors(gt) *etc*. and is not relevant to the choice of reflections for refinement. *R*-factors based on *F*^2^ are statistically about twice as large as those based on *F*, and *R*- factors based on ALL data will be even larger.

### Fractional atomic coordinates and isotropic or equivalent isotropic displacement parameters (Å^2^)

**Table:**

	*x*	*y*	*z*	*U*_iso_*/*U*_eq_	
O1A	0.1984 (3)	0.7390 (2)	0.5535 (2)	0.0159 (6)	
N1A	0.1813 (3)	0.4983 (3)	0.5337 (2)	0.0129 (7)	
C1A	0.2499 (4)	0.8338 (4)	0.5299 (3)	0.0148 (9)	
Pt1	0.453703 (13)	0.146225 (12)	0.137337 (10)	0.00981 (6)	
O1	0.6022 (2)	0.2325 (2)	0.1494 (2)	0.0142 (6)	
N1	0.5504 (3)	−0.0034 (3)	0.1351 (2)	0.0117 (7)	
C1	0.5956 (4)	0.3279 (3)	0.1894 (3)	0.0119 (8)	
Pt2	0.156255 (13)	0.642655 (12)	0.448083 (10)	0.01040 (6)	
O2A	0.1157 (3)	0.7813 (2)	0.3578 (2)	0.0146 (6)	
N2A	0.1119 (3)	0.5367 (3)	0.3526 (2)	0.0139 (7)	
C2A	0.1688 (4)	0.8749 (3)	0.3638 (3)	0.0124 (8)	
N2	0.3162 (3)	0.0506 (3)	0.1192 (2)	0.0117 (7)	
O2	0.3455 (2)	0.2901 (2)	0.1274 (2)	0.0144 (6)	
C2	0.3757 (4)	0.3818 (4)	0.1618 (3)	0.0138 (8)	
C3A	0.2857 (4)	0.8653 (4)	0.4187 (3)	0.0150 (9)	
O3A	0.2689 (3)	0.8974 (3)	0.5925 (2)	0.0224 (7)	
O3	0.6886 (3)	0.3818 (3)	0.1888 (2)	0.0184 (6)	
C3	0.4711 (4)	0.3702 (3)	0.2382 (3)	0.0119 (8)	
O4A	0.1305 (3)	0.9664 (2)	0.3257 (2)	0.0194 (6)	
C4A	0.3951 (4)	0.7854 (4)	0.3772 (3)	0.0160 (9)	
H4AA	0.4094	0.7139	0.4198	0.019*	
H4AB	0.3915	0.7702	0.3067	0.019*	
O4	0.3279 (3)	0.4755 (2)	0.1359 (2)	0.0197 (7)	
C4	0.4412 (4)	0.2950 (4)	0.3374 (3)	0.0144 (8)	
H4A	0.3528	0.2818	0.3524	0.017*	
H4B	0.4929	0.2227	0.3433	0.017*	
C5A	0.4822 (4)	0.8787 (4)	0.3913 (3)	0.0199 (9)	
H5AA	0.5246	0.8649	0.4532	0.024*	
H5AB	0.5415	0.8956	0.3326	0.024*	
C5	0.4845 (4)	0.3897 (4)	0.3947 (3)	0.0194 (9)	
H5A	0.5692	0.3745	0.4149	0.023*	
H5B	0.4262	0.4110	0.4518	0.023*	
O5	0.9600 (3)	0.7036 (3)	0.0445 (3)	0.0242 (7)	
C6A	0.3696 (4)	0.9654 (4)	0.3991 (3)	0.0189 (9)	
H6AA	0.3674	1.0129	0.4560	0.023*	
H6AB	0.3572	1.0130	0.3363	0.023*	
C6	0.4756 (4)	0.4727 (4)	0.2997 (3)	0.0184 (9)	
H6A	0.3996	0.5238	0.3011	0.022*	
H6B	0.5495	0.5162	0.2815	0.022*	
O6	0.8025 (3)	0.5297 (3)	0.0351 (2)	0.0258 (7)	
C7A	0.2141 (4)	0.4841 (4)	0.6263 (3)	0.0165 (9)	
H7AA	0.2312	0.5486	0.6584	0.020*	
C7	0.6712 (4)	−0.0248 (4)	0.1398 (3)	0.0146 (8)	
H7A	0.7204	0.0350	0.1505	0.017*	
O7	0.1062 (3)	0.9814 (3)	0.7410 (2)	0.0262 (8)	
C8	0.7264 (4)	−0.1337 (4)	0.1292 (3)	0.0167 (9)	
H8A	0.8128	−0.1470	0.1325	0.020*	
C8A	0.2239 (4)	0.3760 (4)	0.6776 (3)	0.0224 (10)	
H8AA	0.2466	0.3679	0.7441	0.027*	
O8	0.1100 (3)	0.7860 (3)	0.8755 (3)	0.0251 (7)	
C9A	0.2008 (4)	0.2815 (4)	0.6324 (3)	0.0217 (10)	
H9AA	0.2082	0.2083	0.6675	0.026*	
C9	0.6574 (4)	−0.2223 (4)	0.1142 (3)	0.0167 (9)	
H9A	0.6957	−0.2960	0.1056	0.020*	
C10A	0.1663 (4)	0.2928 (4)	0.5345 (3)	0.0175 (9)	
C10	0.5291 (4)	−0.2017 (3)	0.1117 (3)	0.0140 (8)	
C11A	0.1572 (3)	0.4046 (3)	0.4885 (3)	0.0123 (8)	
C11	0.4803 (4)	−0.0907 (3)	0.1219 (3)	0.0099 (8)	
C12A	0.1200 (3)	0.4261 (3)	0.3902 (3)	0.0122 (8)	
C12	0.3531 (4)	−0.0603 (3)	0.1137 (3)	0.0122 (8)	
C13A	0.0898 (4)	0.3367 (4)	0.3380 (3)	0.0172 (9)	
C13	0.2739 (4)	−0.1442 (4)	0.0995 (3)	0.0151 (9)	
C14A	0.0511 (4)	0.3660 (4)	0.2424 (3)	0.0200 (9)	
H14A	0.0291	0.3086	0.2040	0.024*	
C14	0.1496 (4)	−0.1059 (4)	0.0891 (3)	0.0161 (9)	
H14B	0.0908	−0.1585	0.0805	0.019*	
C15A	0.0451 (4)	0.4774 (4)	0.2046 (3)	0.0202 (10)	
H15A	0.0196	0.4972	0.1397	0.024*	
C15	0.1151 (4)	0.0079 (4)	0.0915 (3)	0.0165 (9)	
H15B	0.0325	0.0345	0.0820	0.020*	
C16A	0.0763 (4)	0.5623 (4)	0.2614 (3)	0.0158 (9)	
H16A	0.0719	0.6393	0.2344	0.019*	
C16	0.1994 (4)	0.0843 (4)	0.1076 (3)	0.0131 (8)	
H16B	0.1732	0.1625	0.1104	0.016*	
C17A	0.1382 (4)	0.2005 (4)	0.4768 (3)	0.0197 (9)	
H17A	0.1466	0.1240	0.5048	0.024*	
C17	0.4475 (4)	−0.2880 (4)	0.1002 (3)	0.0156 (9)	
H17B	0.4782	−0.3650	0.0969	0.019*	
C18A	0.1011 (4)	0.2228 (4)	0.3857 (3)	0.0210 (10)	
H18A	0.0812	0.1615	0.3509	0.025*	
C18	0.3240 (4)	−0.2579 (4)	0.0939 (3)	0.0169 (9)	
H18B	0.2706	−0.3152	0.0855	0.020*	
H5V	0.996 (5)	0.731 (5)	−0.010 (3)	0.05 (2)*	
H5W	0.919 (4)	0.649 (3)	0.036 (4)	0.028 (14)*	
H6V	0.759 (5)	0.526 (6)	−0.012 (3)	0.06 (2)*	
H6W	0.776 (4)	0.477 (3)	0.074 (3)	0.024 (14)*	
H7V	0.038 (3)	0.996 (5)	0.718 (4)	0.038 (16)*	
H7W	0.151 (4)	0.948 (4)	0.698 (3)	0.030 (15)*	
H8V	0.109 (6)	0.843 (4)	0.832 (4)	0.05 (2)*	
H8W	0.177 (3)	0.753 (5)	0.856 (4)	0.042 (17)*	

### Atomic displacement parameters (Å^2^)

**Table:**

	*U*^11^	*U*^22^	*U*^33^	*U*^12^	*U*^13^	*U*^23^
O1A	0.0166 (15)	0.0163 (16)	0.0153 (14)	−0.0036 (12)	−0.0017 (11)	−0.0012 (12)
N1A	0.0088 (16)	0.0150 (19)	0.0144 (17)	−0.0021 (13)	−0.0003 (13)	0.0013 (14)
C1A	0.013 (2)	0.014 (2)	0.017 (2)	0.0004 (16)	−0.0020 (16)	−0.0025 (17)
Pt1	0.00974 (9)	0.00862 (10)	0.01139 (9)	−0.00119 (6)	−0.00273 (6)	−0.00017 (6)
O1	0.0094 (14)	0.0170 (16)	0.0171 (14)	−0.0038 (11)	0.0001 (11)	−0.0043 (12)
N1	0.0152 (17)	0.0113 (18)	0.0082 (15)	−0.0010 (13)	−0.0008 (13)	0.0017 (13)
C1	0.013 (2)	0.015 (2)	0.0076 (18)	−0.0016 (16)	−0.0038 (15)	0.0031 (16)
Pt2	0.01050 (9)	0.00972 (10)	0.01119 (9)	−0.00181 (6)	−0.00188 (6)	−0.00010 (6)
O2A	0.0183 (15)	0.0101 (15)	0.0158 (14)	0.0003 (12)	−0.0065 (11)	0.0017 (11)
N2A	0.0095 (16)	0.0140 (19)	0.0178 (18)	−0.0016 (13)	−0.0003 (13)	0.0008 (14)
C2A	0.015 (2)	0.007 (2)	0.0144 (19)	0.0029 (16)	−0.0002 (16)	−0.0007 (16)
N2	0.0115 (17)	0.0108 (18)	0.0132 (16)	−0.0028 (13)	−0.0016 (13)	−0.0009 (13)
O2	0.0139 (14)	0.0098 (15)	0.0205 (15)	0.0016 (11)	−0.0080 (12)	−0.0010 (12)
C2	0.013 (2)	0.014 (2)	0.015 (2)	−0.0046 (16)	−0.0009 (16)	−0.0032 (17)
C3A	0.019 (2)	0.014 (2)	0.013 (2)	−0.0034 (17)	−0.0032 (16)	−0.0013 (16)
O3A	0.0259 (17)	0.0258 (18)	0.0175 (15)	−0.0096 (14)	0.0030 (13)	−0.0103 (13)
O3	0.0155 (15)	0.0225 (17)	0.0182 (15)	−0.0089 (13)	−0.0013 (12)	−0.0013 (12)
C3	0.0105 (19)	0.009 (2)	0.017 (2)	−0.0030 (15)	−0.0038 (16)	0.0001 (16)
O4A	0.0216 (16)	0.0119 (16)	0.0257 (16)	0.0004 (12)	−0.0080 (13)	−0.0019 (13)
C4A	0.014 (2)	0.020 (2)	0.014 (2)	0.0002 (17)	−0.0005 (16)	−0.0025 (17)
O4	0.0226 (16)	0.0088 (16)	0.0288 (17)	−0.0007 (12)	−0.0121 (13)	0.0030 (12)
C4	0.014 (2)	0.017 (2)	0.0122 (19)	−0.0004 (16)	−0.0015 (16)	0.0007 (16)
C5A	0.018 (2)	0.025 (3)	0.018 (2)	−0.0067 (18)	−0.0035 (17)	0.0012 (18)
C5	0.017 (2)	0.029 (3)	0.013 (2)	0.0005 (18)	−0.0033 (17)	−0.0067 (18)
O5	0.0225 (17)	0.0217 (19)	0.0295 (19)	−0.0084 (14)	0.0011 (14)	−0.0052 (15)
C6A	0.022 (2)	0.017 (2)	0.019 (2)	−0.0072 (18)	−0.0057 (18)	0.0005 (18)
C6	0.020 (2)	0.017 (2)	0.020 (2)	−0.0005 (17)	−0.0083 (18)	−0.0037 (18)
O6	0.0273 (18)	0.027 (2)	0.0249 (18)	−0.0142 (15)	−0.0095 (15)	0.0045 (15)
C7A	0.012 (2)	0.022 (2)	0.016 (2)	−0.0033 (17)	−0.0019 (16)	0.0016 (17)
C7	0.014 (2)	0.016 (2)	0.014 (2)	−0.0013 (16)	−0.0036 (16)	0.0006 (16)
O7	0.0158 (17)	0.044 (2)	0.0187 (17)	0.0070 (15)	−0.0038 (14)	−0.0091 (15)
C8	0.016 (2)	0.019 (2)	0.014 (2)	0.0022 (17)	−0.0025 (16)	0.0017 (17)
C8A	0.017 (2)	0.029 (3)	0.020 (2)	−0.0005 (19)	−0.0056 (18)	0.0079 (19)
O8	0.0190 (18)	0.027 (2)	0.0301 (19)	−0.0022 (15)	0.0001 (14)	−0.0068 (16)
C9A	0.017 (2)	0.017 (2)	0.029 (2)	0.0001 (18)	−0.0030 (18)	0.0074 (19)
C9	0.022 (2)	0.015 (2)	0.0115 (19)	0.0080 (17)	−0.0041 (17)	0.0019 (16)
C10A	0.009 (2)	0.018 (2)	0.023 (2)	−0.0026 (16)	0.0033 (16)	0.0033 (18)
C10	0.021 (2)	0.012 (2)	0.0087 (19)	0.0015 (16)	0.0000 (16)	−0.0003 (15)
C11A	0.0083 (18)	0.007 (2)	0.021 (2)	0.0011 (15)	0.0011 (16)	−0.0002 (16)
C11	0.015 (2)	0.0052 (19)	0.0086 (18)	0.0006 (15)	−0.0012 (15)	0.0012 (14)
C12A	0.0066 (18)	0.015 (2)	0.015 (2)	−0.0026 (15)	0.0013 (15)	−0.0007 (16)
C12	0.015 (2)	0.014 (2)	0.0080 (18)	0.0002 (16)	−0.0008 (15)	−0.0003 (16)
C13A	0.010 (2)	0.017 (2)	0.024 (2)	−0.0034 (16)	0.0036 (17)	−0.0067 (18)
C13	0.019 (2)	0.017 (2)	0.0111 (19)	−0.0051 (17)	−0.0016 (16)	−0.0018 (16)
C14A	0.015 (2)	0.021 (2)	0.026 (2)	−0.0024 (17)	−0.0017 (18)	−0.0123 (19)
C14	0.020 (2)	0.017 (2)	0.013 (2)	−0.0074 (17)	−0.0010 (16)	−0.0027 (16)
C15A	0.017 (2)	0.029 (3)	0.016 (2)	−0.0014 (18)	−0.0031 (17)	−0.0067 (19)
C15	0.010 (2)	0.026 (3)	0.014 (2)	−0.0007 (17)	−0.0029 (16)	−0.0034 (17)
C16A	0.013 (2)	0.019 (2)	0.016 (2)	−0.0009 (17)	−0.0011 (16)	−0.0015 (17)
C16	0.014 (2)	0.013 (2)	0.0121 (19)	0.0029 (16)	−0.0009 (15)	−0.0017 (16)
C17A	0.0064 (19)	0.019 (2)	0.031 (2)	−0.0013 (16)	0.0015 (17)	0.0135 (19)
C17	0.026 (2)	0.012 (2)	0.0102 (19)	−0.0051 (17)	0.0010 (17)	−0.0039 (16)
C18A	0.015 (2)	0.013 (2)	0.036 (3)	−0.0026 (17)	0.0033 (19)	−0.0094 (19)
C18	0.024 (2)	0.013 (2)	0.015 (2)	−0.0076 (17)	−0.0043 (17)	−0.0015 (17)

### Geometric parameters (Å, º)

**Table:**

O1A—C1A	1.294 (5)	C6—H6A	0.9900
O1A—Pt2	2.012 (3)	C6—H6B	0.9900
N1A—C7A	1.328 (5)	O6—H6V	0.84 (2)
N1A—C11A	1.368 (5)	O6—H6W	0.83 (2)
N1A—Pt2	1.989 (3)	C7A—C8A	1.400 (6)
C1A—O3A	1.225 (5)	C7A—H7AA	0.9500
C1A—C3A	1.534 (6)	C7—C8	1.394 (6)
Pt1—N1	1.992 (3)	C7—H7A	0.9500
Pt1—N2	1.996 (3)	O7—H7V	0.83 (2)
Pt1—O2	2.000 (3)	O7—H7W	0.83 (2)
Pt1—O1	2.010 (3)	C8—C9	1.378 (6)
O1—C1	1.290 (5)	C8—H8A	0.9500
N1—C7	1.333 (5)	C8A—C9A	1.373 (7)
N1—C11	1.366 (5)	C8A—H8AA	0.9500
C1—O3	1.240 (5)	O8—H8V	0.85 (2)
C1—C3	1.524 (5)	O8—H8W	0.84 (2)
Pt2—N2A	1.996 (3)	C9A—C10A	1.402 (6)
Pt2—O2A	2.000 (3)	C9A—H9AA	0.9500
O2A—C2A	1.303 (5)	C9—C10	1.409 (6)
N2A—C16A	1.330 (5)	C9—H9A	0.9500
N2A—C12A	1.359 (5)	C10A—C11A	1.410 (6)
C2A—O4A	1.217 (5)	C10A—C17A	1.466 (6)
C2A—C3A	1.532 (6)	C10—C11	1.393 (6)
N2—C16	1.328 (5)	C10—C17	1.434 (6)
N2—C12	1.349 (5)	C11A—C12A	1.422 (6)
O2—C2	1.296 (5)	C11—C12	1.423 (5)
C2—O4	1.224 (5)	C12A—C13A	1.400 (6)
C2—C3	1.528 (5)	C12—C13	1.403 (6)
C3A—C6A	1.541 (6)	C13A—C14A	1.404 (6)
C3A—C4A	1.560 (6)	C13A—C18A	1.440 (6)
C3—C6	1.538 (6)	C13—C14	1.415 (6)
C3—C4	1.560 (5)	C13—C18	1.419 (6)
C4A—C5A	1.548 (6)	C14A—C15A	1.367 (6)
C4A—H4AA	0.9900	C14A—H14A	0.9500
C4A—H4AB	0.9900	C14—C15	1.374 (6)
C4—C5	1.545 (6)	C14—H14B	0.9500
C4—H4A	0.9900	C15A—C16A	1.400 (6)
C4—H4B	0.9900	C15A—H15A	0.9500
C5A—C6A	1.544 (6)	C15—C16	1.385 (6)
C5A—H5AA	0.9900	C15—H15B	0.9500
C5A—H5AB	0.9900	C16A—H16A	0.9500
C5—C6	1.548 (6)	C16—H16B	0.9500
C5—H5A	0.9900	C17A—C18A	1.331 (6)
C5—H5B	0.9900	C17A—H17A	0.9500
O5—H5V	0.85 (2)	C17—C18	1.379 (6)
O5—H5W	0.84 (2)	C17—H17B	0.9500
C6A—H6AA	0.9900	C18A—H18A	0.9500
C6A—H6AB	0.9900	C18—H18B	0.9500
			
C1A—O1A—Pt2	121.2 (2)	H6AA—C6A—H6AB	111.1
C7A—N1A—C11A	118.6 (4)	C3—C6—C5	89.3 (3)
C7A—N1A—Pt2	128.4 (3)	C3—C6—H6A	113.8
C11A—N1A—Pt2	112.9 (3)	C5—C6—H6A	113.8
O3A—C1A—O1A	122.3 (4)	C3—C6—H6B	113.8
O3A—C1A—C3A	120.5 (4)	C5—C6—H6B	113.8
O1A—C1A—C3A	117.2 (3)	H6A—C6—H6B	111.0
N1—Pt1—N2	81.97 (13)	H6V—O6—H6W	100 (6)
N1—Pt1—O2	173.29 (12)	N1A—C7A—C8A	121.2 (4)
N2—Pt1—O2	92.66 (12)	N1A—C7A—H7AA	119.4
N1—Pt1—O1	93.57 (12)	C8A—C7A—H7AA	119.4
N2—Pt1—O1	174.96 (12)	N1—C7—C8	120.9 (4)
O2—Pt1—O1	91.63 (11)	N1—C7—H7A	119.6
C1—O1—Pt1	122.9 (2)	C8—C7—H7A	119.6
C7—N1—C11	118.9 (3)	H7V—O7—H7W	107 (5)
C7—N1—Pt1	128.1 (3)	C9—C8—C7	120.9 (4)
C11—N1—Pt1	112.9 (3)	C9—C8—H8A	119.5
O3—C1—O1	120.5 (4)	C7—C8—H8A	119.5
O3—C1—C3	121.7 (4)	C9A—C8A—C7A	120.4 (4)
O1—C1—C3	117.8 (3)	C9A—C8A—H8AA	119.8
N1A—Pt2—N2A	82.27 (14)	C7A—C8A—H8AA	119.8
N1A—Pt2—O2A	174.10 (12)	H8V—O8—H8W	99 (6)
N2A—Pt2—O2A	93.23 (12)	C8A—C9A—C10A	120.2 (4)
N1A—Pt2—O1A	93.17 (13)	C8A—C9A—H9AA	119.9
N2A—Pt2—O1A	175.17 (12)	C10A—C9A—H9AA	119.9
O2A—Pt2—O1A	91.21 (11)	C8—C9—C10	118.9 (4)
C2A—O2A—Pt2	120.2 (2)	C8—C9—H9A	120.6
C16A—N2A—C12A	119.2 (4)	C10—C9—H9A	120.6
C16A—N2A—Pt2	128.1 (3)	C9A—C10A—C11A	115.9 (4)
C12A—N2A—Pt2	112.6 (3)	C9A—C10A—C17A	126.4 (4)
O4A—C2A—O2A	122.4 (4)	C11A—C10A—C17A	117.6 (4)
O4A—C2A—C3A	120.5 (4)	C11—C10—C9	117.1 (4)
O2A—C2A—C3A	117.0 (3)	C11—C10—C17	119.1 (4)
C16—N2—C12	119.0 (3)	C9—C10—C17	123.8 (4)
C16—N2—Pt1	128.0 (3)	N1A—C11A—C10A	123.7 (4)
C12—N2—Pt1	113.0 (3)	N1A—C11A—C12A	115.8 (4)
C2—O2—Pt1	121.2 (2)	C10A—C11A—C12A	120.6 (4)
O4—C2—O2	121.5 (4)	N1—C11—C10	123.2 (4)
O4—C2—C3	120.3 (3)	N1—C11—C12	115.6 (3)
O2—C2—C3	118.2 (4)	C10—C11—C12	121.1 (4)
C2A—C3A—C1A	108.4 (3)	N2A—C12A—C13A	123.0 (4)
C2A—C3A—C6A	116.8 (3)	N2A—C12A—C11A	116.4 (3)
C1A—C3A—C6A	113.5 (3)	C13A—C12A—C11A	120.5 (4)
C2A—C3A—C4A	116.7 (3)	N2—C12—C13	123.7 (4)
C1A—C3A—C4A	111.6 (3)	N2—C12—C11	116.5 (3)
C6A—C3A—C4A	88.8 (3)	C13—C12—C11	119.7 (4)
C1—C3—C2	109.7 (3)	C12A—C13A—C14A	116.7 (4)
C1—C3—C6	114.4 (3)	C12A—C13A—C18A	118.1 (4)
C2—C3—C6	116.1 (3)	C14A—C13A—C18A	125.2 (4)
C1—C3—C4	109.0 (3)	C12—C13—C14	116.0 (4)
C2—C3—C4	117.5 (3)	C12—C13—C18	118.5 (4)
C6—C3—C4	88.8 (3)	C14—C13—C18	125.5 (4)
C5A—C4A—C3A	88.0 (3)	C15A—C14A—C13A	119.9 (4)
C5A—C4A—H4AA	114.0	C15A—C14A—H14A	120.1
C3A—C4A—H4AA	114.0	C13A—C14A—H14A	120.1
C5A—C4A—H4AB	114.0	C15—C14—C13	119.3 (4)
C3A—C4A—H4AB	114.0	C15—C14—H14B	120.4
H4AA—C4A—H4AB	111.2	C13—C14—H14B	120.4
C5—C4—C3	88.5 (3)	C14A—C15A—C16A	120.2 (4)
C5—C4—H4A	113.9	C14A—C15A—H15A	119.9
C3—C4—H4A	113.9	C16A—C15A—H15A	119.9
C5—C4—H4B	113.9	C14—C15—C16	120.6 (4)
C3—C4—H4B	113.9	C14—C15—H15B	119.7
H4A—C4—H4B	111.1	C16—C15—H15B	119.7
C6A—C5A—C4A	89.2 (3)	N2A—C16A—C15A	121.0 (4)
C6A—C5A—H5AA	113.8	N2A—C16A—H16A	119.5
C4A—C5A—H5AA	113.8	C15A—C16A—H16A	119.5
C6A—C5A—H5AB	113.8	N2—C16—C15	121.3 (4)
C4A—C5A—H5AB	113.8	N2—C16—H16B	119.3
H5AA—C5A—H5AB	111.0	C15—C16—H16B	119.3
C4—C5—C6	89.0 (3)	C18A—C17A—C10A	120.7 (4)
C4—C5—H5A	113.8	C18A—C17A—H17A	119.7
C6—C5—H5A	113.8	C10A—C17A—H17A	119.7
C4—C5—H5B	113.8	C18—C17—C10	119.2 (4)
C6—C5—H5B	113.8	C18—C17—H17B	120.4
H5A—C5—H5B	111.0	C10—C17—H17B	120.4
H5V—O5—H5W	112 (6)	C17A—C18A—C13A	122.4 (4)
C3A—C6A—C5A	88.9 (3)	C17A—C18A—H18A	118.8
C3A—C6A—H6AA	113.8	C13A—C18A—H18A	118.8
C5A—C6A—H6AA	113.8	C17—C18—C13	122.3 (4)
C3A—C6A—H6AB	113.8	C17—C18—H18B	118.8
C5A—C6A—H6AB	113.8	C13—C18—H18B	118.8

### Hydrogen-bond geometry (Å, º)

**Table:**

*D*—H···*A*	*D*—H	H···*A*	*D*···*A*	*D*—H···*A*
O5—H5*W*···O6	0.84	1.97	2.802 (5)	170
O6—H6*W*···O3	0.83	2.03	2.842 (4)	165
O7—H7*W*···O3*A*	0.83	1.93	2.753 (4)	169
O8—H8*V*···O7	0.85	1.95	2.806 (5)	175
O5—H5*V*···O8^i^	0.85	1.98	2.821 (5)	169
O6—H6*V*···O4^ii^	0.84	2.00	2.839 (4)	173
O7—H7*V*···O4*A*^iii^	0.83	2.00	2.828 (4)	176
O8—H8*W*···O1^iv^	0.84	2.43	3.125 (4)	142
O8—H8*W*···O3^iv^	0.84	2.17	2.971 (4)	160
C7*A*—H7*AA*···O3^iv^	0.95	2.55	3.376 (5)	146
C7—H7*A*···O7^iv^	0.95	2.49	3.145 (5)	126
C8—H8*A*···O5^v^	0.95	2.59	3.259 (6)	128
C9—H9*A*···O6^v^	0.95	2.51	3.436 (6)	165
C14*A*—H14*A*···O8^vi^	0.95	2.35	3.230 (6)	154
C14—H14*B*···O5^vii^	0.95	2.36	3.309 (6)	174
C18*A*—H18*A*···O4*A*^v^	0.95	2.38	3.196 (5)	144
C18—H18*B*···O4^v^	0.95	2.55	3.151 (5)	121

Symmetry codes: (i) *x*+1, *y*, *z*−1; (ii) −*x*+1, −*y*+1, −*z*; (iii) −*x*, −*y*+2, −*z*+1; (iv) −*x*+1, −*y*+1, −*z*+1; (v) *x*, *y*−1, *z*; (vi) −*x*, −*y*+1, −*z*+1; (vii) *x*−1, *y*−1, *z*.

## References

[bb1] Agilent (2012). *CrysAlis PRO* Agilent Technologies Ltd, Santa Clara, CA, USA.

[bb2] Brandenburg, K. (2011). *DIAMOND* Crystal Impact GbR, Bonn, Germany.

[bb3] Ferreira, A. D. Q., Bino, A. & Gibson, D. (1997). *Inorg. Chim. Acta*, **265**, 155–161.

[bb4] Sheldrick, G. M. (2008). *Acta Cryst.* A**64**, 112–122. 10.1107/S0108767307043930 18156677

[bb5] Tu, C., Lin, J., Shao, Y. & Guo, Z. (2003). *Inorg. Chem.* **42**, 5795–5797. 10.1021/ic034604q 12971744

[bb6] Tu, C., Wu, X., Liu, C., Wang, X., Xu, Q. & Guo, Z. (2004). *Inorg. Chim. Acta*, **357**, 95–102.

[bb7] Westrip, S. P. (2010). *J. Appl. Cryst.* **43**, 920–925.

[bb8] Yoo, J., Sohn, Y. S. & Do, Y. (1999). *J. Inorg. Biochem.* **73**, 187–193. 10.1016/s0162-0134(99)00016-1 10331247

